# Supplementary material for: SyNDI: synchronous network data integration framework
Source: BMC Bioinformatics. 2018 Nov 6;19:403. doi: 10.1186/s12859-018-2426-5 (PMC6219086; doi:10.1186/s12859-018-2426-5)
Supplement: Supplementary file 5 — Files of the example presented in Additional file 4. This additional file is a zip package that contains all files used in Additional file 4. Detailed descriptions of each of these files are presented in a README file included in this zip package. (GZ 30411 kb) [file 12859_2018_2426_MOESM5_ESM.gz › SyNDITools/Meme2Fimo/meme_4.9.0_4.tar.gz!/meme_4.9.0/website/html/mast-faq.html]

MAST - FAQ


|  |  |
| --- | --- |
|  | MEME Suite The MEME Suite web application requires the use of JavaScript   Javascript doesn't seem to be available on your browser.  Frequently Asked Questions     ---    - **Q.** MAST returns "Your sequence contains   unrecognized characters..."  ---     - **Q.** MAST returns "Your sequence contains unrecognized   characters..." **A.** The file must be a **plain text** file. MSWord   files, for example, are **not** acceptable. MSWord files can be   converted to plain text by:    "File"-->"Save As"-->"Save As Type"-->"Plain Text'   (\*.txt). |
